# Increasing fault tolerance of data plane on the internet of things using the software-defined networks

**DOI:** 10.7717/peerj-cs.543

**Published:** 2021-05-27

**Authors:** Katayoun Bakhshi Kiadehi, Amir Masoud Rahmani, Amir Sabbagh Molahosseini

**Affiliations:** 1 Department of Computer Engineering, Islamic Azad University Kerman Branch, Kerman, Iran; 2 Future Technology Research Center, National Yunlin University of Science and Technology, Douliou, Yunlin, Taiwan

**Keywords:** Software-defined network, Internet of things, Data plane, Fault tolerance, SDN, IoT, Openflow

## Abstract

Considering the Internet of Things (IoT) impact in today’s world, uninterrupted service is essential, and recovery has received more attention than ever before. Fault-tolerance (FT) is an essential aspect of network resilience. Fault-tolerance mechanisms are required to ensure high availability and high reliability in systems. The advent of software-defined networking (SDN) in the IoT plays a significant role in providing a reliable communication platform. This paper proposes a data plane fault-tolerant architecture using the concepts of software-defined networks for IoT environments. In this work, a mathematical model called Shared Risk Link Group (SRLG) calculates redundant paths as the primary and backup non-overlapping paths between network equipment. In addition to the fault tolerance, service quality was considered in the proposed schemes. Putting the percentage of link bandwidth usage and the rate of link delay in calculating link costs makes it possible to calculate two completely non-overlapping paths with the best condition. We compare our two proposed dynamic schemes with the hybrid disjoint paths (Hybrid_DP) method and our previous work. IoT developments, wireless and wired equipment are now used in many industrial and commercial applications, so the proposed hybrid dynamic method supports both wired and wireless devices; furthermore multiple link failures will be supported in the two proposed dynamic schemes. Simulation results indicate that, while reducing the error recovery time, the two proposed dynamic designs lead to improved service quality parameters such as packet loss and delay compared to the Hybrid_DP method. The results show that in case of a link failure in the network, the proposed hybrid dynamic scheme’s recovery time is approximately 12 ms. Furthermore, in the proposed hybrid dynamic scheme, on average, the recovery time, the packet loss, and the delay improved by 22.39%, 8.2%, 5.66%, compared to the Hybrid_DP method, respectively.

## Introduction

Due to advancements in technology and the impacts of the IoT on everyday life, the number of devices providing various Internet services increased. It is estimated that over 50 billion devices will be connected to the internet by 2025 (Ericsson, 2018). In recent years, communication has attracted the attention of many researchers (Mohd Aman et al., 2020). These developments must be considered in-network redesign (Hakiria et al., 2014). As the communication network becomes an important element of the system, its performance, reliability, and resilience should be guaranteed at all times (Duran, Leal & Botero, 2017). Generally, fault-tolerance is an essential part of the design of any network. To overcome these network service issues, fault-tolerance mechanisms are essential to identify and heal the network failures (Rehman, Aguiar & Barraca, 2019). Link failure become a significant problem that should be considered in today’s networks (Sgambelluri et al., 2013; Bellagamba, Kempf & Skoldstrom, 2011), as it can lead to congestion throughout the network and reduce network efficiency (Qin et al., 2014). Easy failure detection and a quick recovery mechanism are crucial for network systems. The traditional networking infrastructure consists of various networking devices such as switches, routers, and intermediate devices pre-programmed with complex rules. Due to the resource-constrained features of the devices, they cannot be pre-programmed with multiple rules to provide optimal network services. Therefore, traditional network technologies cannot handle IoT requirements efficiently to provide scalability, seamlessly, and cost-effectively (Bera, Misra & Vasilakos, 2017; Davoli et al., 2018; Zhang et al., 2018; Tomovic et al., 2016). Because of the wide variety of IoT and its organizational complexity, the SDN-based IoT architecture has been proposed for effective management (Darabseh & Freris, 2017; Jararweh et al., 2018; Haque et al., 2019; Alam et al., 2019).

The IoT architecture has improved using novel technologies such as SDN (Javed et al., 2018). By separating the control plane from the data plane, SDN has changed network switches into simple devices, which only perform forwarding packets according to the control Plane commands (Fonseca & Mota, 2017; Xia et al., 2015; Betts et al., 2014; Nunes et al., 2014). The network control Plane is implemented in a centralized central controller. Since the controller has a global view of the network and is aware of the latest network topology, the fault detection and recovery processes are simpler than traditional networks (Jain & Paul, 2013; Foerster, Schmid & Vissicchio, 2018; Rehmani et al., 2019; Kreutz, Ramos & Verissimo, 2013; Kreutz et al., 2015). Applying SDN, configuration and management can be reduced greatly; furthermore, SDN can build up a more tight association inside the IoT environment (Bilal, Faiz & Shah, 2017).

In this study, we mainly focus on applying SDN technology in the IoT environment. The SDN architecture consists of three levels and interfaces between planes (standardized Application Programmable Interfaces (APIs)) (Rangisetti & Tamma, 2017; Zahmatkesh & Kunz, 2017):

- Data plane: The data plane includes routers, switches, access points (AP), and IoT devices that can be dynamically configured and programmed. At this level, switching, routing, and implementing policies implemented by the control plane are performed. In addition, the data plane is responsible for forwarding packets.
- Control plane: The control plane is responsible for deciding how packets must be handled and forwarded to the network devices. The Control plane provides a holistic view of the network. It has all the information related to the data plane equipment, capabilities, and network topology. The most popular controllers in the software-defined networks are OpenDaylight (ODL) (OpenDaylight, 2020), Floodlight (Floodlight, 2020), Beacon (Erickson, 2013), open network operating system (ONOS) (Berde et al., 2014), POX (OpenFlow, 2018).
- Application plane: The applications and services that define network behavior reside in this plane. The application plane is used for creating new rules using APIs for certain types of incoming packets that are passed to the controller when needed.
- Southbound interface (SBI): This interface is a communication interface between the control plane and the data plane. OpenFlow (OF) (OpenFlow, 2014) is a default standard protocol for this communication. Other protocols and/or APIs for southbound interface are: Open virtual switch database (OVSDB) (Pfaff & Davie, 2013), forwarding and control element separation (ForCES) (Haleplidis et al., 2015), and protocol oblivious forwarding (POF) (Softbank Hikari, 2013), network configuration protocol (NETCONF) (Enns et al., 2011).
- Northbound interface (NBI): This interface (Cox et al., 2017) is responsible for all communication and interactions between the control plane and the application plane. This interface helps to program the controller. Famous northbound interfaces include Representational State Transfer Application Programming Interface (Rest API), Programming languages, high-level programming language (e.g., Frenetic, Procera, etc.).

There is a possibility of fault in any of the three planes of an SDN (Yu et al., 2018; Malik et al., 2019) that, in this study, link failure in the data plane has been considered. One of the most common methods used in link failure recovery is calculating the backup path and sending it through this path (Kreutz et al., 2015; Chen et al., 2015). One of the criteria for choosing a backup path is choosing the shortest path with the least cost (Ramos, Martinello & Esteve Rothenberg, 2013). In this paper, the SRLG mathematical model is used to calculate two non-overlapping paths, which will be installed two paths as the primary and backup non-overlapping paths between the source and destination (Oki, 2012). In case of a failure in the main path, the backup path will immediately replace, to prevent irreparable damages.

The main contributions of this paper are as follows:

- The proposed architecture includes redundant paths between (1) the OpenFlow switches; (2) the access points and the switches.
- We have proposed the wired dynamic scheme and the hybrid dynamic design.
- The main and backup paths are calculated at the beginning and duration of the simulation.
- The two proposed dynamic methods support multiple link failures.

The rest of this paper is structured as follows. First, the literature review on fault tolerance in the SDN and SDN-based IoT is presented in “Literature Review”. Then, in “Methodology”, the proposed architecture and designs are described in detail. Next, in “Simulation Setting”, the simulation settings will be presented, and simulation results will be evaluated. Finally, “Conclusion” concludes this paper.

## Literature Review

Increasing the SDN's fault tolerance is a new research direction for researchers, so there is limited literature on improving fault tolerance SDN architecture (Berde et al., 2014). The usability of the SDN technology was already proved in various fields. In this respect, many studies target the link failures inside the SDN and the recovery mechanisms. Many studies discuss the vision, the architecture, and the challenging problems related to IoT (Gubbi et al., 2013; Borgia, 2014). But the whole concept of SDN-based IoT is in its infancy, and standardization efforts are still underway. The most integration of SDN and IoT domains is in the cellular network, IoT management, and security. It is worth noting that most of these studies are not experimentally validated (Tayyaba et al., 2017). In this section, some articles and research in SDN-based IoT are presented.

Savas et al. (2018) proposed a recovery-aware switch controller assignment and routing (RASCAR) algorithm, enabling fast recovery after failures. This work showed, more effective switch-controller assignment and control-path routing strategies can reduce the control-path restoration delay. Besides significantly reduces the recovery time of affected data paths. Cheng et al. (2017) proposed a new resilience approach to balance failure recovery time and forwarding rule occupation. At first, the problem is formulated in the proposed scheme as an integer linear programming model.

An efficient heuristic named the congestion-aware local fast reroute (CALFR) was then designed. Baddeley et al. (2019) have presented Atomic-SDN, a highly reliable and low-latency solution for SDN in low-power wireless. Atomic-SDN introduces a novel synchronous flooding (SF) architecture capable of dynamically configuring SF protocols to satisfy complex SDN control requirements. The proposed scheme improves the SDN controller by latency, reliability, and energy-efficiency metrics. Chattopadhyay et al. (2020) have proposed Aloe, an auto-scalable, distributed, and fault-tolerant SDN control plane using “self-stabilizing” controller over an IoT platform.

In this paper, a distributed SDN control plane is integrated with the in-network processing infrastructure, such that the control plane can dynamically maintaining a fault-tolerant architecture. The proposed framework ensures the availability and significant reduction in flow-setup delay by deploying instances in the vicinity of the resource constraint IoT devices dynamically. Sinh et al. (2018) have introduced an SDN/NFV architecture for deploying IoT framework. IoT applications are deployed as SDN controller applications on top of SDN networks to meet the requirements of deploying IoT services from different providers and recover an IoT service in case of failure. Wu et al. (2018) have proposed a method called UbiFlow to maintain scalability and network stability in the IoT based on multi-control SDNs. One of the problems of the proposed method is overheads. This work improved mobility management, handover optimization, and access point selection functions from the IoT devices to controllers. Zhang et al. (2018) proposed a flexible flow aggregation method named Forwarding Rule Multiplexing (FRM) to minimize the total number of forwarding rules in SDN-based IoT. This work multiplex, different traffic flows traverses through the same path into an aggregated flow with the VLAN-ID label.

Furthermore, this method extends to SDN protection against link failure and reduces backup path forwarding rules. Huang, Wang & Wu (2018) proposed software define networks and wireless distribution system (SDWDS), which assisted fault recovery automation scheme. In SDWDS, packets can be directly and wirelessly forwarded between two APs in case of the Ethernet link failure at one of the APs. The SDWDS was considered for automatic fault recovery in IoT environments, which support more than one Ethernet link failure in the network. Huang, Chung & Liu (2018) have proposed a Stay Connected (SCONN) mechanism with dual-band wireless networking technology. When the controller detects the abnormal situation between an AP and the control system with SCONN, packets will be adaptively rerouted through the backup wireless mesh network consisting of dual-band APs. SCONN integrates dual-band wireless networking, the IEEE 802.11s and SDN aiming to dynamically utilize the available bandwidth in the network for data transmission in abnormal situations. Bakhshi Kiadehi, Rahmani & Sabbagh Molahosseini (2021) have used a mathematical model called SRLG to calculate non-overlapping paths for increasing data plane fault tolerance. In this method, the Fault Tolerance bundle executes the algorithm at the beginning of the simulation to calculate the primary and backup path. The proposed scheme reduces the error recovery time and improves service quality parameters such as packet loss, delay, and packet jitter—this work support single link failure.

Overall, it can be seen that the SDN's effectiveness in the fault tolerance discussion has recently attracted researchers' attention to improve fault tolerance in the SDN-based IoT. However, only (Bakhshi Kiadehi, Rahmani & Sabbagh Molahosseini, 2021) considered fault tolerance along with the quality of service. Also, the two main and backup paths do not have links with shared risk. Therefore, despite the (Bakhshi Kiadehi, Rahmani & Sabbagh Molahosseini, 2021) method's benefits, it is possible to retrieve a single link failure. Besides, our previous work wired switch was considered. Two non-overlapping paths as the primary and backup non-overlapping paths are calculated at the simulation’s beginning. The network changes over the simulation duration, so calculated paths may not have the best quality. Therefore, this work solves the weakness of the previous work.

On the other hand, the proposed method supports multiple link failures, and a combination of wired and wireless switches is considered. Besides, the paths are calculated at the simulation's beginning and duration and calculate the primary and backup path. Therefore, if a more optimal path is found, the traffic is cut from the current path and sent again from the new optimal path. So if a link fails, data not be lost.

## Methodology

**Figure 1:**
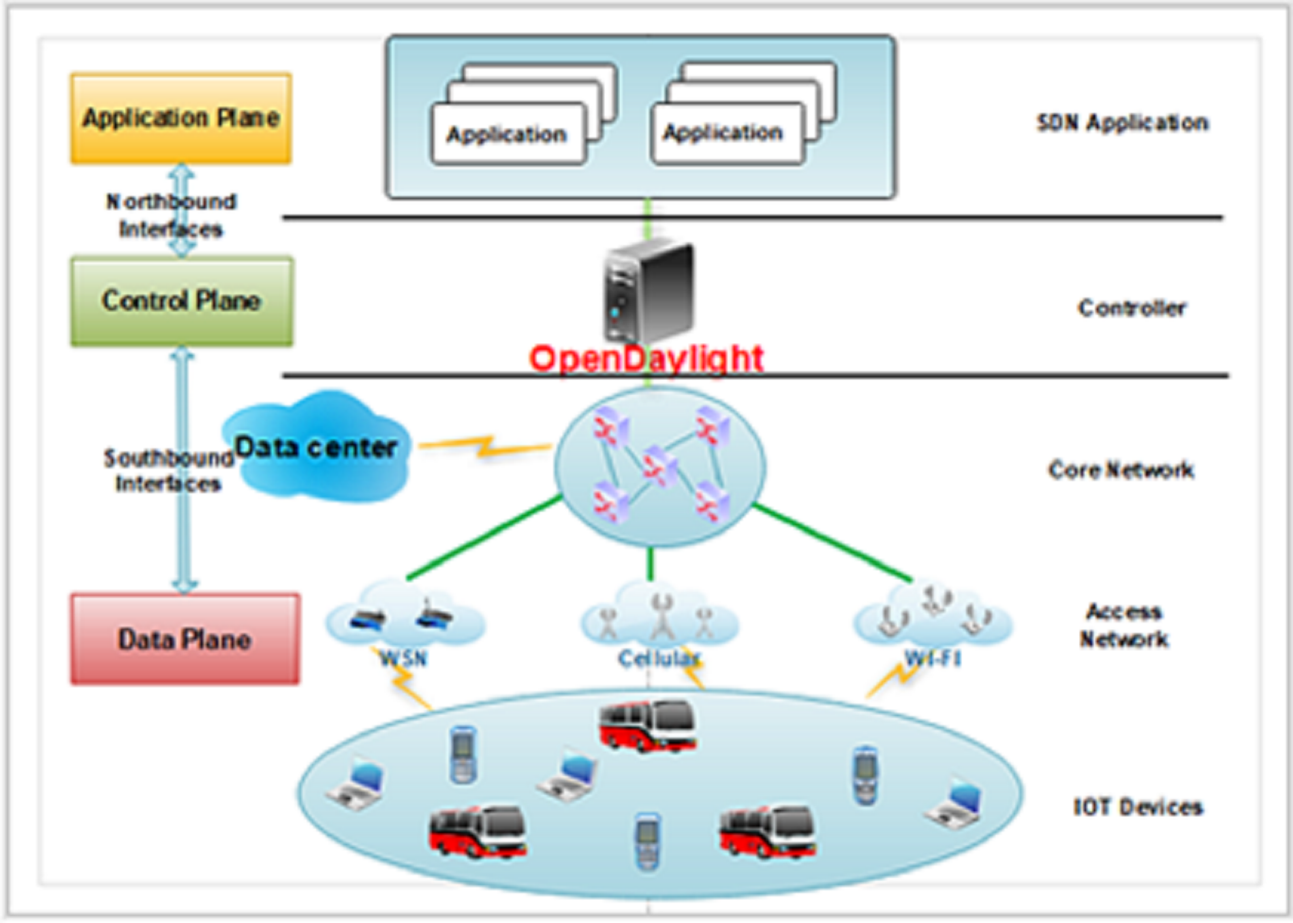
The proposed architecture.

The proposed architecture provides a data plane fault-tolerant feature. Figure 1 shows the proposed architecture’s general view (Farris et al., 2019). The proposed SRLG algorithm implemented at the control plane will be introduced later. At this plane, there is an ODL controller that is responsible for controlling the network. There are OpenFlow switches, AP, IoT devices, and other network equipment at the data plane. As shown in Fig. 1, the SDN paradigm can be implemented at various places, such as data center, core, and access network. This study provides a method to increase data plane fault tolerance. The scope of this work is core and access network, including a connection between (1) the OpenFlow switches; (2) the access points and the switches. Two dynamic methods are proposed, which will be explained in “Designs”. The primary purpose of this work is to support multiple link failures. The secondary goal is to support a combination of wired and wireless devices.

### The connection between network devices

Failure of the link between network devices interferes with the transmission of network traffic. The proposed SRLG algorithm is implemented in the control plane to increase data plane fault tolerance. Then the controller will install two non-overlapping paths on the devices. The two paths do not have links with shared risk (Li et al., 2012). The two dynamic schemes have been proposed for these connections, and detail of these methods will be described in “Designs”.

### Designs

This work is developed by inspiration from our previous work (Bakhshi Kiadehi, Rahmani & Sabbagh Molahosseini, 2021) that proposed static design for wired switches. The proposed static design supported single link failure. The IoT consists of many intelligent objects that interact through many interconnected wired and wireless networks (Asghari, Rahmani & Javadi, 2018; Macedo et al., 2019). So in this work, we proposed a hybrid dynamic design that is considered a combination of wired and wireless devices. The two proposed dynamic designs, the Hybrid_DP method and the static scheme, shown in Table 1, are explained.

**Table 1:** The specifications of the designs.

Number	Name of design	The method of calculating the backup path	Failure	Switches
1	Static (Hakiri et al., 2015)	At the beginning of the simulation	Single link failure	Wired
2	Hybrid_DP	At the beginning of the simulation	Single link failure	Wired and wireless
3	Wired Dynamic	At the beginning and in the duration of the simulation	Multiple link failure	Wired
4	Hybrid Dynamic	At the beginning and in the duration of the simulation	Multiple link failure	Wired and wireless

#### Static design

In this method, the Fault Tolerance bundle executes the proposed SRLG algorithm at the beginning of the simulation and calculates the primary and backup path. In case of failure of the main path, the traffic is sent through the backup path. In this case, the wired switches are considered. The two non-overlapping paths which are calculated in the proposed SRLG algorithm support single link failure. In our previous work (Bakhshi Kiadehi, Rahmani & Sabbagh Molahosseini, 2021), we assumed two links as a main and backup path; it is possible to increase these two paths to N paths in the proposed SRLG algorithm. However, this number should be specified in the proposed SRLG algorithm at the beginning of the simulation and cannot be changed in the simulation duration.

#### Hybrid_DP design

This method is one of the standard methods for calculating two disjoint paths used by various works (Katoh & Kumar, 2011). This method can significantly increase the fault tolerance associated with link failures by calculating disjoint paths. Two disjoint paths are two paths from source to destination without any shared link in a directed graph (Benson et al., 2018; Liao & Tsai, 2018; Abe, Mantar & Yayimli, 2015). In the DP method in our previous work (Bakhshi Kiadehi, Rahmani & Sabbagh Molahosseini, 2021) wired switches were considered. The Hybrid_DP method is similar to the wired DP method, but the proposed method considers a combination of wired and wireless switches.

#### Wired dynamic design

In this method, the Fault Tolerance bundle executes the proposed SRLG algorithm at the simulation’s beginning and duration and calculates the primary and backup path. Since non-overlapping is considered in choosing the primary and backup path, it is necessary to recalculate both primary and backup paths every T-second. With the proposed wired dynamic scheme, it is possible to recover multiple link failures. In such a case, the wired switches are considered.

#### Hybrid dynamic design

This method is similar to the proposed wired dynamic scheme. Because in real networks, wireless and wired switches are used simultaneously, so in the proposed hybrid dynamic method, a combination of wired and wireless switches is considered. This design supports multiple link failures.

### The SRLG algorithm

**Figure 2:**
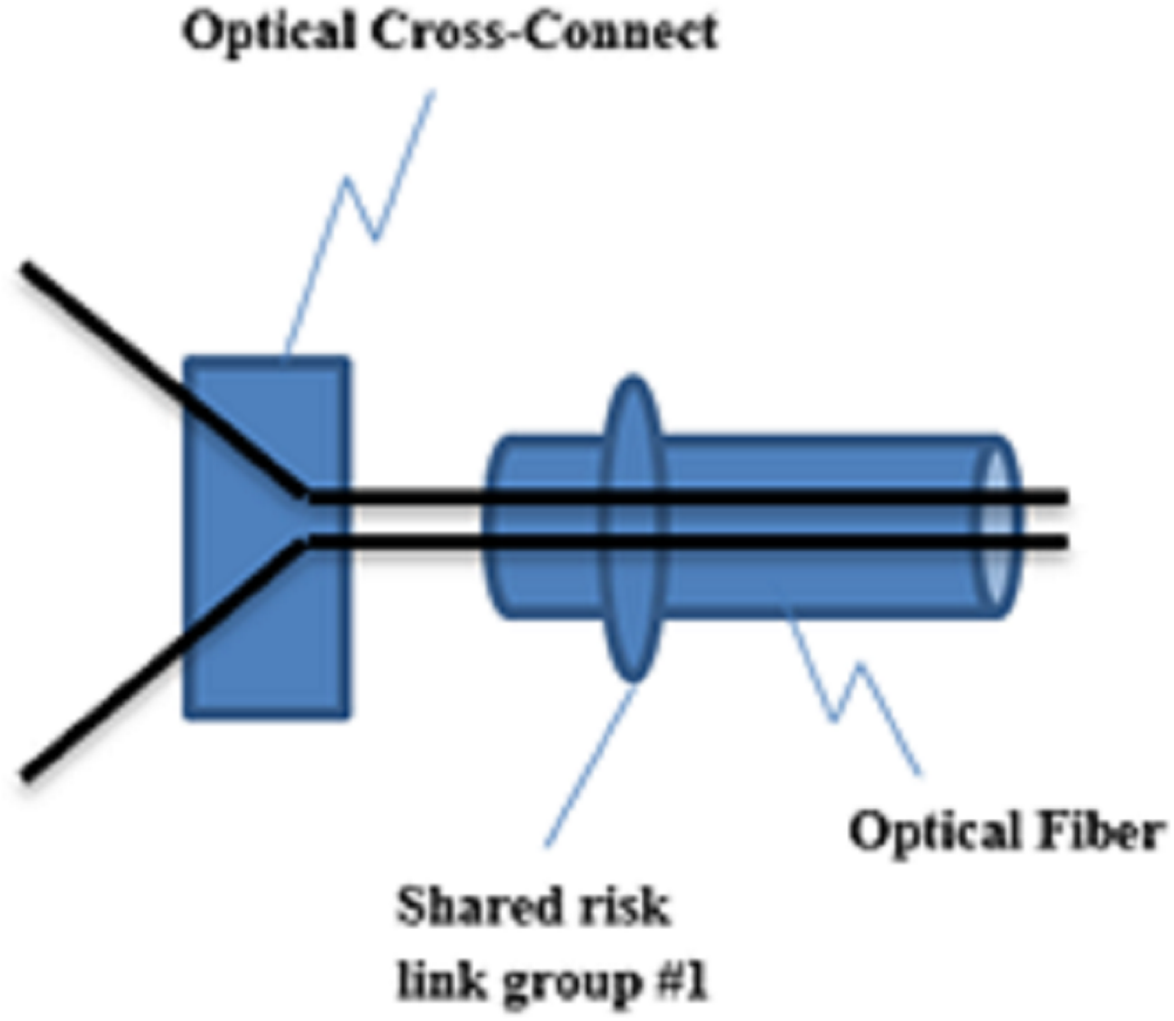
A group of links to show SRLG concept.

As shown in Fig. 2 (Bakhshi Kiadehi, Rahmani & Sabbagh Molahosseini, 2021), SRLG is defined as a group of links that are affected simultaneously when a network failure occurs. Links in an optical network may use a shared resource, such as a duct or conduit. The failure of this resource results in the simultaneous failure of multiple links. Such failures are referred to as SRLG failure (Ahuja, Ramasubramanian & Krunz, 2011). When multiple links are passed through a physical channel, the channel failure causes simultaneous failures of multiple links.

**Figure 3:**
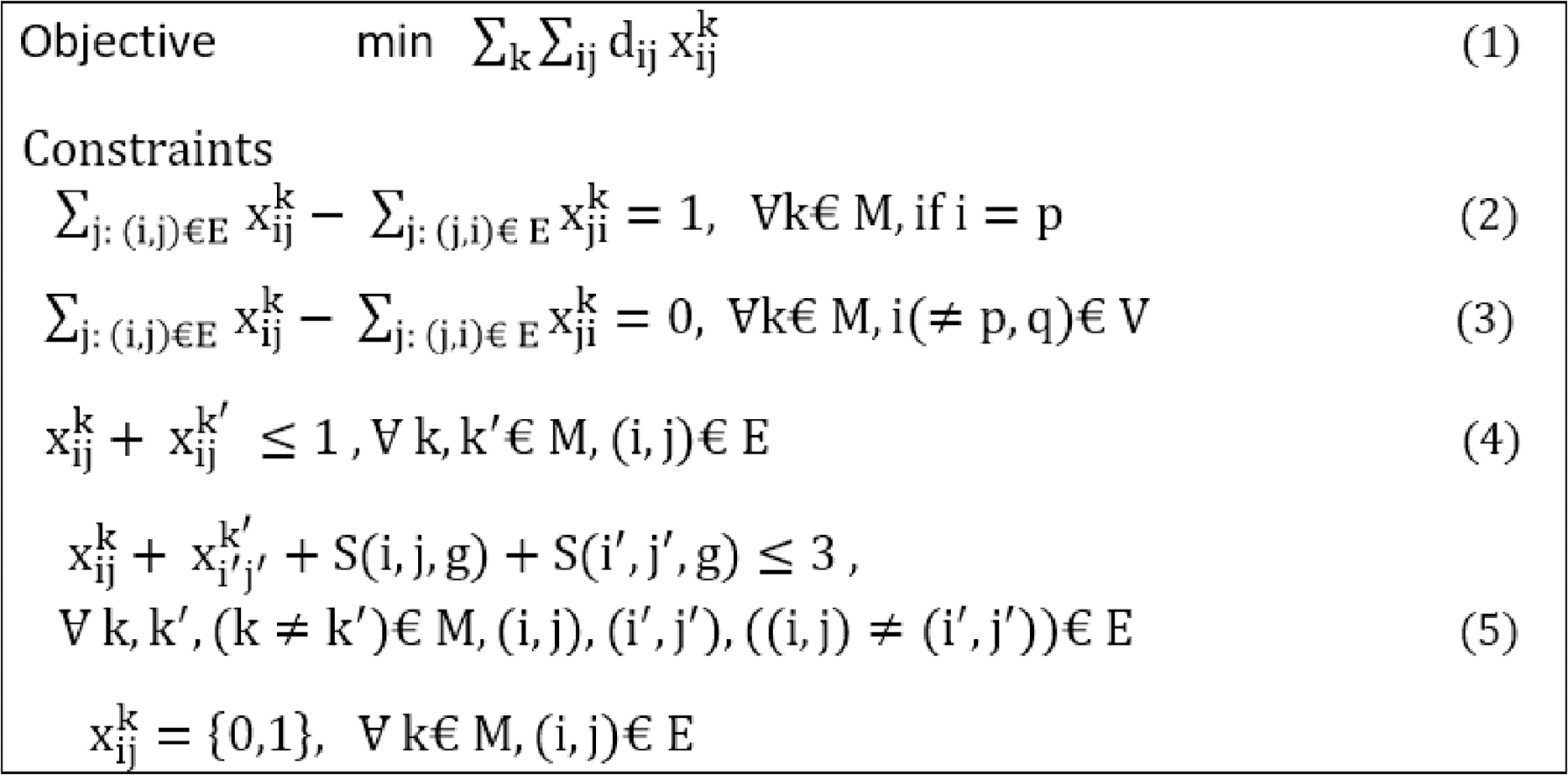
Linear Programming constraints of SRLG algorithm.

By modeling a network in the form of the proposed SRLG algorithm and working it out, several non-overlapping paths that have no shared risk links can be calculated. In the proposed architecture, this model is used to calculate the main and backup paths. The ILP relationships in Fig. 3 (Bakhshi Kiadehi, Rahmani & Sabbagh Molahosseini, 2021) describe the SRLG model used in the proposed architecture (Mininet, 2020).

Equation (1) is an objective function that calculated the non-overlapping paths at the lowest cost, from node i to node j. Equations (2)–(5) are the constraints. Equations (2) and (3) state the flow conservation conditions, which calculate the differences between input and output traffic volume. Respectively, if it is zero, i.e., the edge is not on the path, and if it is one, it indicates that the edge is on the path. Equation (4) controls the number of shared risk links. Equation (5) determines how many shared risk links can exist, resulting in the absence of shared risk links in the paths.

The network of a directed graph G (V, E) is considered. E is the set of network links, and V the set of network nodes, forming the network topology graph, which are the OpenFlow switches in the proposed architecture. A link from node i to node j is denoted by (i. j), which belongs to E. The variables i and j are used to refer to links, and the variables k presented the number of the non-overlapping paths that calculate in our proposed designs. The variable G is presented as the number of shared risk links are considered in proposed topologies. The variable $x_{ij}^{\;}$ indicates the presence or absence of a link in the calculated path. Besides, the $d_{ij}^{\;}$ Variable is also the cost of each link. Finally, the variable M determines the total number of non-overlapping paths.

In this work, the quality of services is considered in addition to fault tolerance. For this purpose, the variable $d_{ij}$ in the proposed SRLG algorithm is determined based on Eq. (6). The variable $u_{ij\;}$ indicates the percentage of bandwidth link usage, and $p_{ij\;}$ represents the rate of link delay. Putting these two parameters in the calculation of link costs makes it possible to calculate two completely overlapping paths with the least end-to-end delay and the degree of link congestion. As a result, a reduction occurs in the packet delay and loss for network traffic flows. Parameter α is considered a regulatory parameter to the share of delay and the percentage of link usage in calculating the cost. In this study, both parameters are equally considered. But based on the different services, the importance of each of these parameters can be differently considered. Since the type and quality of links in networks can influence the quality of services, the coefficient w makes wired links cost less than wireless links. In other words, the proposed SRLG algorithm assigns a lower cost to wired links to compare to wireless links to priorities a path with wired links.

(6) $$d_{ij}=w\left(\alpha u_{ij}+\left(1-\alpha \right)p_{ij}\right)$$

#### Implementing the SRLG algorithm

In the two proposed dynamic designs, the proposed SRLG algorithm is implemented as a fault tolerance (F-T) module or bundle in the controller. This bundle should collect information about the switches and APs, before solving the SRLG algorithm. For this purpose, it is necessary to interact with other bundles in the controller such as topology manager, switch manager, statistics manager, forwarding rules manager, flow manager, Dijkstra's bundle.

The F-T module obtains information through the switch manager, topology manager, and statistics manager modules. Based on the obtained information, the proposed SRLG algorithm computes the primary and backup paths. If the F-T module fails to calculate two non-overlapping paths and does not answer the SRLG algorithm, it will be called the Dijkstra bundle for computing paths. Finally, this path is installed on the switches by calling the flow manager module (Bakhshi Kiadehi, Rahmani & Sabbagh Molahosseini, 2021).

**Figure 4:**
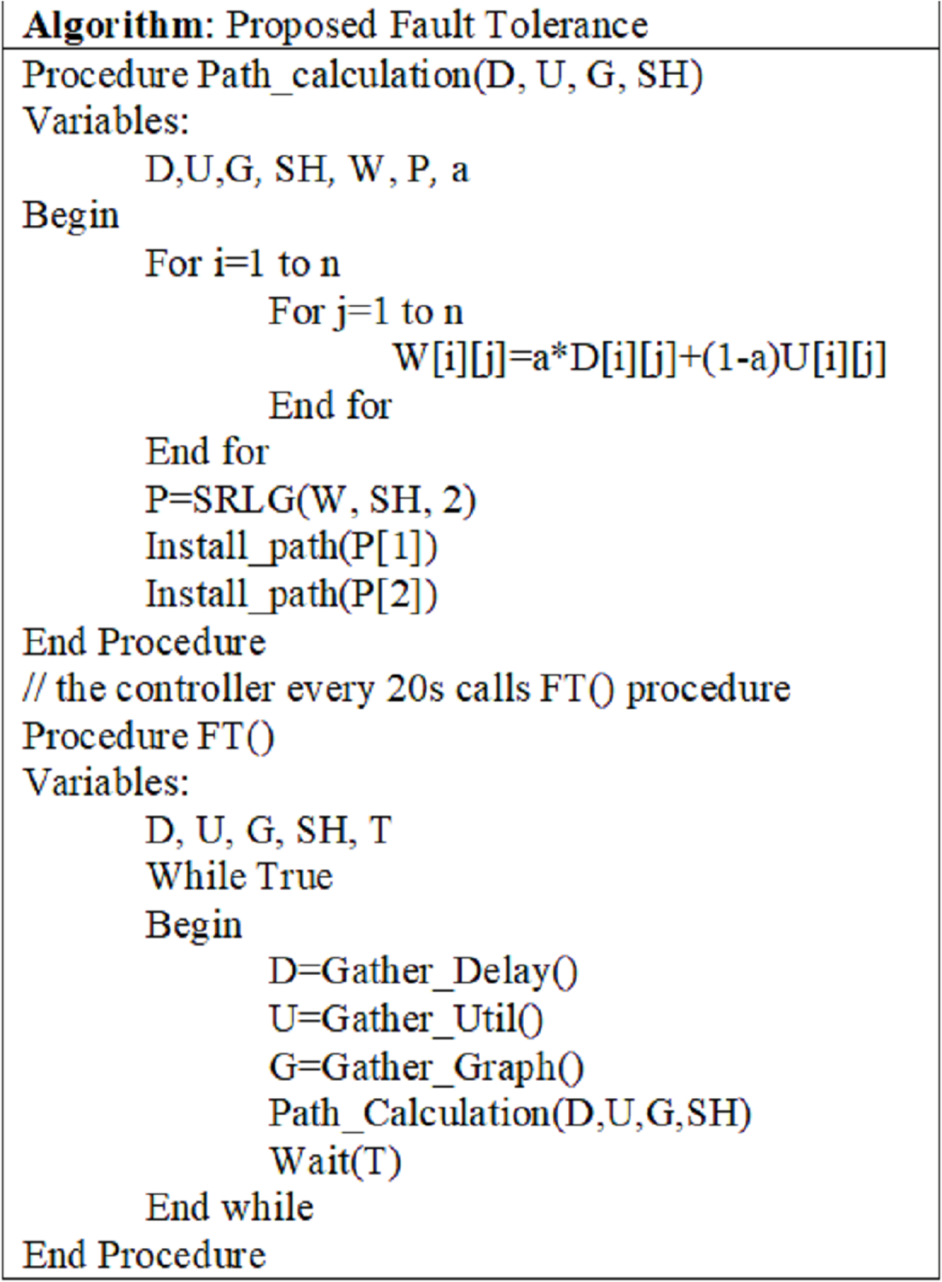
Pseudo-Code of the SRLG algorithm.

Figure 4 (Bakhshi Kiadehi, Rahmani & Sabbagh Molahosseini, 2021) shows the pseudo-code, which is the proposed F-T module. In this pseudo-code, the Path_Calculation () function performs the path calculation and installation operation. This function's output is non-overlapping paths installed on the switches and APs by calling the Install_path () function. The Path_Calculation () function itself is called the F-T () function.

## Simulation Setting

In the two proposed dynamic designs, the primary and backup paths are calculated dynamically at the beginning and duration of the simulation to recover multiple link failures. The two proposed dynamic designs were simulated on the HP ProLiant G9 server with an 8 core CPU and 64G RAM.

The controllers were set up on Ubuntu and the same server. Three different topologies with different switches and AP are considered for all designs presented in Table 1, explained in “Topologies and Simulation Configurations”. These topologies are implemented using Python. The famous OpenDayLight controller is used for the control plane, widely used in commercial and academic fields. The proposed SRLG algorithm is implemented through the Java programming language and added to the OpenDayLight controller as a bundle form. The Mininet-WiFi simulator (Huang, Chung & Liu, 2018) was used to simulate the data Plane for the following reasons:

- As explained in the introduction, this research's work area is the core and access network, and all of these devices are supported by the Mininet-WiFi simulator.
- This work has been developed by inspiration from our previous work (Bakhshi Kiadehi, Rahmani & Sabbagh Molahosseini, 2021). Evaluating the proposed method in the previous work has done with the Mininet-WiFi simulator. For comparison and fair assessment of our proposed methods with the proposed design in (Bakhshi Kiadehi, Rahmani & Sabbagh Molahosseini, 2021), the same environment and simulator were used.
- The Mininet-WiFi simulator is very similar to the natural environment.
- In many research in the SDN-based IoT, the Mininet-WiFi simulator was used (Sinh et al., 2018; Zhang et al., 2018).

### Topologies and simulation configurations

**Figure 5:**
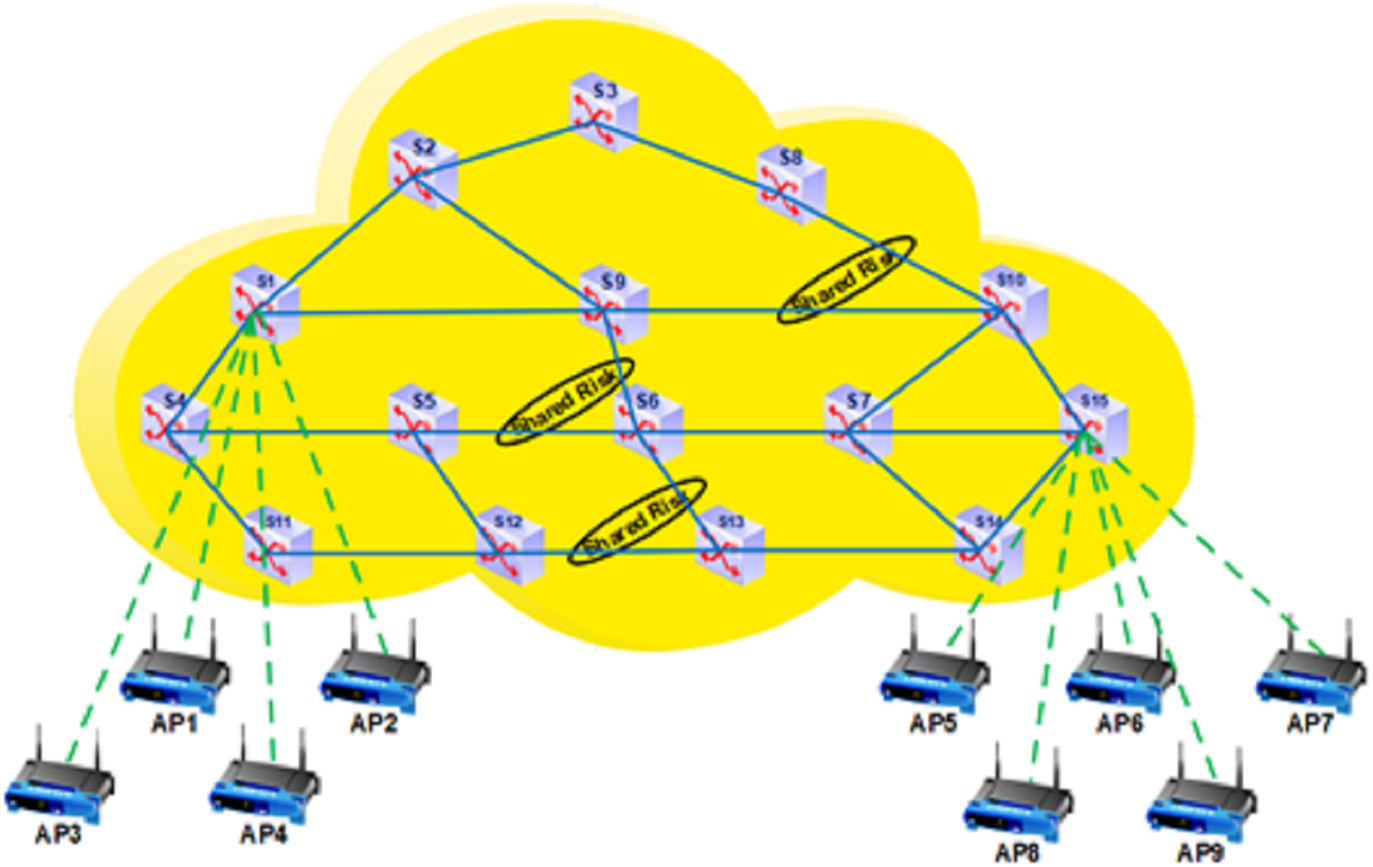
The specification of medium topology.

- Since it is challenging to obtain the data of general IoT topologies (Gubbi et al., 2013; Zhang et al., 2018), we create three topologies in the Mininet-WiFi simulator. Table 2 shows the configurations related to simulation, and Table 3 displays the specifications of each simulated topology.
- The number of traffic flows: traffic flows between network devices. For example, as shown in Fig. 5, in the medium topology, there are seven traffic flows to be deployed in the network.
- The number of shared risk links group: SRLG is defined as a group of links that are affected simultaneously when a network failure occurs. G represents the number of these groups. As shown in Fig. 5, G for medium topology was considered three.
- As shown in Table 2, each topology can be configured with six modes. First, the primary path is disconnected for 2 ms every 20 s during the simulation period. Then, the main path becomes out of reach for 4 ms every 20 s, and it continues is done for 6, 8, 10, and 12 ms.
- TCP incurs substantial overhead over wireless channels during congestion control (Hakiri et al., 2015). Therefore, UDP was used in our work due to its low overhead, latency, and connectionless properties, as shown in Table 2. In our work, to address the particular issues related to reliable transport, the paths are recalculated at T-second intervals in the two proposed dynamic methods. If a link fails or a data packet does not reach the destination for any reason, its delay will be longer than the computed route in the next T-second interval. Therefore, in the proposed SRLG algorithm, a more efficient path is found, the traffic is cut off from the current path and sent again from the new optimal path, and data is not lost.

**Table 2:** The configurations related to simulation.

Parameter	Value
Simulation time	100 s
link failure time (6 modes)	2,4,6, 8,10,12 ms
Link failure intervals	Once every 20 s
Type of sent traffic	UDP: 2 Mbps
Number of executions of each topology	Ten times

**Table 3:** The specifications of simulation topologies.

Name of topology	Number of switches	Number of AP	Number of shared risk link groups	Number of traffic flows
Small	5	3	1	3
Medium	15	9	3	7
Large	30	14	4	16

### Evaluation criteria

All of the designs shown in Table 1, based on the following parameters, have been evaluated.

- *End-to-end delay:* The time between sending the packet from the source to receiving it at the destination includes: processing delay, transfer delay, release delay, and packet delay in the switch queue, which will be described in “End-to-end Delay”. The delay in each step is calculated according to Eq. (7) (Bakhshi Kiadehi, Rahmani & Sabbagh Molahosseini, 2021).

(7) dnodal = dproc + dqueue + dtrans + dprop

- *Packet loss:* The ratio of the number of packets not received at the destination to the total sent packets at the source will be described in “Packet Loss”. Packet loss is expressed as a percentage and is obtained according to Eq. (8) (Bakhshi Kiadehi, Rahmani & Sabbagh Molahosseini, 2021).

(8) $$PLR=\left(1-\frac{Received\;Packets}{Total\;Sent\;Packet}\right)*100$$

- *Average recovery loss:* The average failure detection time and the secondary path's replacement will be described in “Average Recovery Time”.

#### End-to-end delay

As shown in Table 1, in this paper, two dynamic designs have proposed. The Hybrid_DP method has the most delay compare to the two proposed schemes and static design. Furthermore, the static design has less delay compared to the two proposed dynamic schemes.

**Figure 6:**
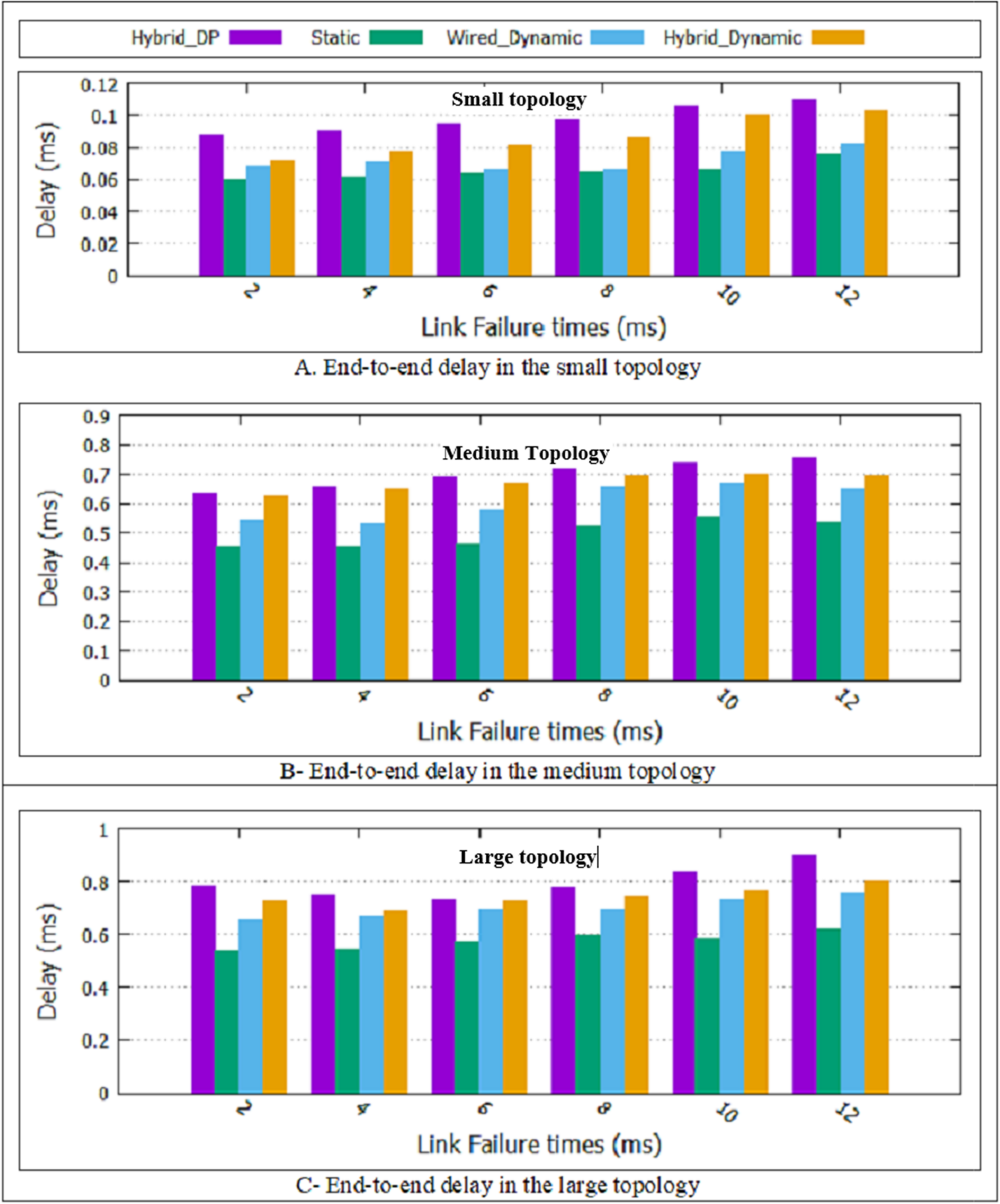
End-to-End delay for all topologies. (A) End-to-end delay in the small topology; (B) end-to-end delay in the medium topology; (C) end-to-end delay in the large topology.

According to the results presented in Fig. 6, it is observed that the end-to-end delay of the two proposed schemes and the static scheme is less than the Hybrid_DP method. The reason is that the proposed SRLG algorithm calculates the main and backup paths. As explained in “The SRLG algorithm”, the variable $d_{ij}$ in the SRLG algorithm is a function of the delay and the percentage of bandwidth use. Therefore, the obtained paths will have a shorter end-to-end delay. In calculating two disjoint paths, the Hybrid_DP method has no consideration of each link's delay, so maybe chosen paths with more end-to-end delay.

In the two proposed dynamic methods, the paths are recalculated at T-second intervals. Since non-overlapping is considered in choosing the primary and backup path, it is necessary to recalculate both primary and backup paths every T-second. Therefore, if a more optimal path is found, the traffic is cut from the current path and sent again from the new optimal path. As a result, two proposed dynamic schemes have a more end-to-end delay compare to the static scheme. The wireless channel has unstable and unreliable conditions compared to the wired mode. Besides, noise and environmental factors have caused the wired dynamic design of less delay than the Hybrid dynamic design.

Finally, a hybrid dynamic scheme can support wired and wireless switches and multiple link failure. As shown in Fig. 6, the hybrid dynamic scheme’s average delay improvement is approximately 5/66% compared to the Hybrid_DP method. In “The Rate of Improvement”, more details will be explained.

#### Packet loss

**Figure 7:**
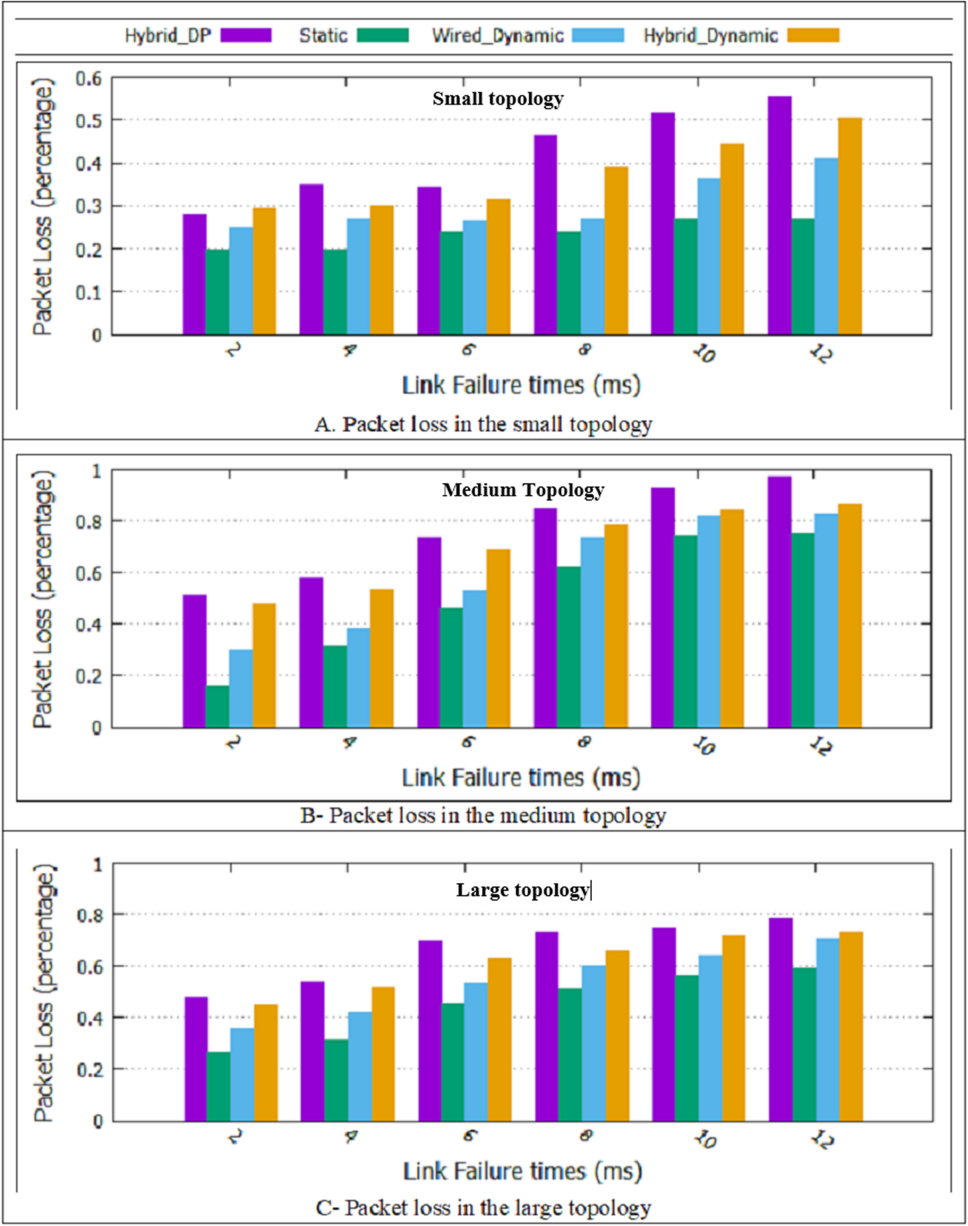
Packet loss for all topologies. (A) A packet loss in the small topology; (B) packet loss in the medium topology; (C) packet loss in the large topology.

As shown in Fig. 7, in all the proposed schemes presented in Table 1, packet loss increases link failure duration increases. The reason is that when the links are failed for a long time due to existing multiple traffic flows in the network, and more packets are lost. Furthermore, the two proposed dynamic designs and the static method have better performance than the Hybrid_DP method in packet loss. Besides, as displayed in Fig. 7, the static method has a minor packet loss than the two proposed dynamic methods. In the two proposed dynamic methods, the paths are recalculated at T-second intervals. Since non-overlapping is considered in choosing the primary and backup path, it is necessary to recalculate both primary and backup paths every T-second. Therefore, if a more optimal path is found, the traffic is cut from the current path and sent again from the new optimal path, which increases packet loss. Finally, the hybrid dynamic scheme has more packet loss than the wired dynamic scheme. Due to noise and environmental factors, the wireless channel has unstable and unreliable conditions compared to the wired mode.

As shown in Fig. 7, the two proposed dynamic schemes and the static method had a more favorable result than the Hybrid_DP method. Compared to the Hybrid_DP method, the average rate of the packet loss improvement in the hybrid dynamic scheme is approximately 8/2%. In “The Rate of Improvement”, computing the percentage of improvement will be explained and detailed.

#### Average recovery time

**Figure 8:**
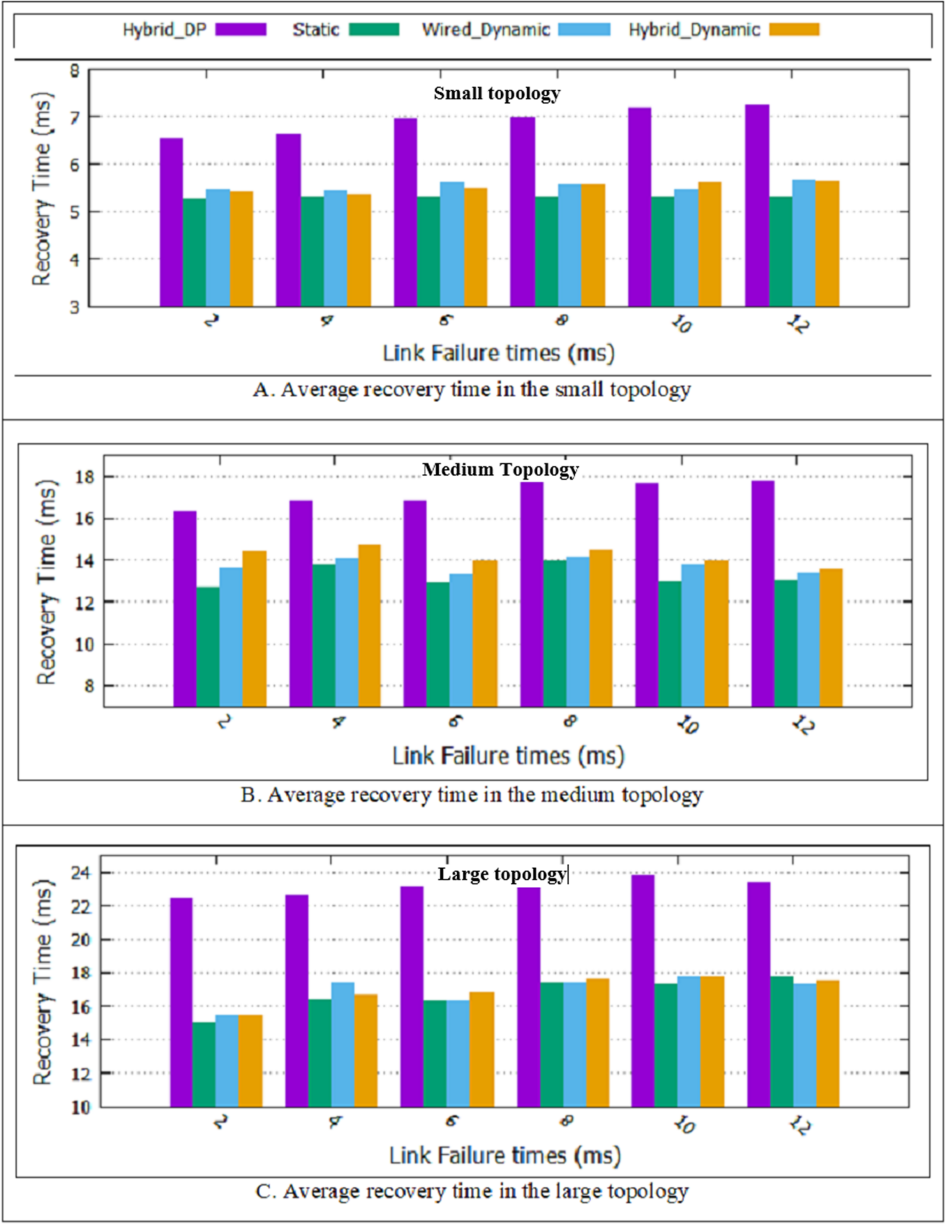
Average recovery time for all topologies. (A) Average recovery time in the small topology; (B) average recovery time in the medium topology; (C) average recovery time in the large topology.

As shown in Table 1, in this paper, two dynamic designs have presented. According to the results presented in Fig. 8, it is observed that the average recovery time of the two proposed dynamic schemes and static method is less than the Hybrid_DP design. It is because the Hybrid_DP method calculates the two non-overlapping paths and ignores QoS parameters. The choice of two non-overlapping paths with high delays will lead to more recovery time. Besides, the two proposed dynamic designs and the static method approximately have the same error recovery time. All three designs have a backup path, and in case of the link failure, the backup path replaces immediately.

The hybrid dynamic scheme, a combination of wired and wireless switches, and multiple link failure are considered. As shown in Fig. 8, the two proposed dynamic schemes and static method had a more favorable result than the Hybrid_DP method. Compared to the Hybrid_DP method, the average recovery time improvement rate in the hybrid dynamic scheme is approximately 22/39%. “The Rate of Improvement”, explains computing the percentage of improvement. The static scheme supports single link failure. In the two dynamic schemes, it is possible to recover multiple link failures. Table 4 shows the experimental results for the two proposed dynamic schemes’, the static design and the Hybrid_DP method for three topologies and on average.

**Table 4:** Summary of simulation results.

Name of topology	Name of scheme	Delay Time (ms)	Packet loss rate	Average recovery time (ms)
	Static (Ericsson, 2018)	0.07	0.23	5.29
	Hybrid_DP method	0.10	0.42	6.91
Small	Wired Dynamic	0.07	0.30	5.54
	Hybrid Dynamic	0.09	0.37	5.49
	Static (Ericsson, 2018)	0.50	0.51	13.24
	Hybrid_DP method	0.70	0.76	17.18
Medium	Wired Dynamic	0.60	0.60	13.74
	Hybrid Dynamic	0.67	0.70	14.18
	Static (Ericsson, 2018)	0.58	0.45	16.68
	Hybrid_DP method	0.80	0.66	23.08
Large	Wired Dynamic	0.70	0.54	16.94
	Hybrid Dynamic	0.74	0.62	16.95

#### The rate of improvement

Our two proposed dynamic schemes can support multiple link failure. The results show that in the case of a link failure in the network, the recovery time in the hybrid dynamic design is approximately 12 ms, which is presented in Table 5. Furthermore, in our scheme, on average, the recovery time, the packet loss, and the delay improved by 22.39%, 8.2%, 5.66%, compared to the Hybrid_DP method, respectively.

**Table 5:** Rate of improvement results.

Methods	Static (Average)	Hybrid_DP (Average)	Wired dynamic (Average)	Hybrid dynamic (Average)	Improvement the hybrid dynamic compare to the Hybrid_DP method (%)	Improvement the static compare to the hybrid dynamic method (%)
Parameters						
Delay	0.38	0.53	0.45	0.5	5.66	24
Packet loss	0.39	0.61	0.48	0.56	8.2	30.35
Recovery time	11.74	15.72	12.07	12.20	22.39	3.77

In the two proposed dynamic methods, the paths are recalculated at T-second intervals. Since non-overlapping is considered in choosing the primary and backup path, it is necessary to recalculate both primary and backup paths every T-second. Therefore, if a more optimal path is found, the traffic is cut from the current path and sent again from the new optimal path, which increases delay, packet loss, and recovery time. It is observed that due to the addition of the path computation at T-second intervals, in the Hybrid Dynamic scheme, on average, delay, packet loss, and recovery time decline by 24%, 30.35%, 3.77%, compared to the static method, respectively. It is suggested that a solution to improve packet loss in this method be considered in future works.

The rate of improvement has been expressed by percentages and will obtain based on Eqs. (9) and (10), and Table 5. Equation (9) (Bakhshi Kiadehi, Rahmani & Sabbagh Molahosseini, 2021) explains the hybrid dynamic scheme’s improvement rate compared to the Hybrid_DP method, and Eq. (10) explains the Static scheme’s improvement rate compared to the Hybrid Dynamic method

(9) Improvement= ((Hybrid_DP - Hybrid Dynamic)/ Hybrid_DP)*100

(10) Improvement= ((Hybrid Dynamic - Static)/ HybridDynamic)*100

## Conclusion

In this paper, the proposed FT-SDN architecture provides data plane fault-tolerant features in the SDN-based IoT. A mathematical model called Shared Risk Link Group (SRLG) calculates redundant paths as the main and backup non-overlapping paths between network equipment, a type of protection failure recovery method. In the proposed methods, in addition to fault tolerance, the quality of service is also considered so that IoT traffic flows are less delayed and the packet is less lost. Furthermore, two proposed dynamic designs calculate the primary and backup paths at the beginning and duration of the simulation, support multiple link failures.

The simulation results indicate that, while reducing the error recovery time, the two proposed dynamic designs lead to improved service quality parameters such as packet loss and delay compared to the Hybrid_DP method. The results show that the proposed hybrid dynamic scheme’s recovery time is approximately 12 ms in case of a link failure in the network. Furthermore, in the proposed hybrid dynamic scheme, on average, the recovery time, the packet loss, and the delay improved by 22.39%, 8.2%, 5.66%, compared to the Hybrid_DP method, respectively. Therefore, according to the obtained results, the proposed hybrid dynamic methods in fault-sensitive IoT environments can be helpful in failure detection and recovery and increase fault tolerance. The static method, the Hybrid dynamic scheme, and wired dynamic design are valid for fault-sensitive IoT environments. Despite the Static scheme’s limitations, in the Hybrid Dynamic scheme, on average, delay, packet loss, recovery time decline by 24%, 30.35%, 3.77%, compared to the Static method, respectively. So, according to the priority of delay, packet loss, or recovery, single or multiple link failure, wire or wireless devices can be chosen as an appropriate design for presenting service in the IoT environment. Due to the added path computation at T-second intervals to support multiple link failure, it is suggested that a solution be considered for optimal packet loss in future work.

## Supplemental Information

10.7717/peerj-cs.543/supp-1 Supplemental Information 1 Code.

10.7717/peerj-cs.543/supp-2 Supplemental Information 2 SRLG.

## Additional Information and Declarations

### Data Availability

The following information was supplied regarding data availability: JAVA code is available in the Supplemental File.

## References

[ref-1] Abe JO, Mantar HA, Yayimli AG (2015). k- Maximally disjoint path routing algorithms for SDN.

[ref-2] Ahuja SS, Ramasubramanian S, Krunz M (2011). SRLG failure localization in optical networks. IEEE/ACM Transactions on Networking.

[ref-3] Alam I, Sharif K, Li F, Latif Z, Karim MM, Nour B, Biswas S, Wang Y (2019). Iot virtualization: a survey of software definition & function virtualization techniques for internet of things. http://arxiv.org/abs/1902.10910.

[ref-4] Asghari P, Rahmani AM, Javadi HHS (2018). Service composition approaches in IoT: a systematic review. Journal of Network and Computer Applications.

[ref-5] Baddeley M, Raza U, Stanoev A, Oikonomou G, Nejabati R, Sooriyabandara M, Simeonidou D (2019). Atomic-SDN: is synchronous flooding the solution to software-defined networking in IoT?. IEEE Access.

[ref-38] Bakhshi Kiadehi K, Rahmani AM, Sabbagh Molahosseini A (2021). A fault-tolerant architecture for internet-of-things based on software-defined networks. Telecommunication Systems.

[ref-6] Bellagamba E, Kempf J, Skoldstrom P (2011). Link failure detection and traffic redirection in an OpenFlow network, US Patent 8,665,699. https://patents.google.com/patent/US20110286324A1/en.

[ref-7] Benson KE, Wang G, Venkatasubramanian N, Kim Y-J (2018). Ride: a resilient IoT data exchange middleware leveraging SDN and edge cloud resources.

[ref-8] Bera S, Misra S, Vasilakos AV (2017). Software-defined networking for Internet of Things: a survey. IEEE Internet of Things Journal.

[ref-9] Berde P, Gerola M, Hart J, Higuchi Y, Kobayashi M, Koide T, Lantz B, O'Connor B, Radoslavov P, Snow W, Parulkar G (2014). Onos: towards an open, distributed SDN OS.

[ref-10] Betts M, Fratini S, Davis N, Dolin R (2014). SDN architecture.

[ref-11] Bilal T, Faiz Z, Shah MA (2017). Software defined networks: an analysis on robust security practices.

[ref-12] Borgia E (2014). The internet of things vision: key features, applications and open issues. Computer Communications.

[ref-13] Chattopadhyay S, Chatterjee S, Nandi S, Chakraborty S (2020). Aloe: fault-tolerant network management and orchestration framework for IoT applications. IEEE Transactions on Network and Service Management.

[ref-14] Chen J, Chen J, Xu F, Yin M, Zhang W (2015). When software defined networks meet fault tolerance: a survey.

[ref-15] Cheng Z, Zhang X, Li Y, Yu S, Lin R, He L (2017). Congestion-aware local reroute for fast failure recovery in software-defined networks. IEEE/OSA Journal of Optical Communications and Networking.

[ref-16] Cox JH, Chung J, Donovan S, Ivey J, Clark RJ, Riley G, Owen HL (2017). Advancing software-defined networks: a survey. IEEE Access.

[ref-17] Darabseh A, Freris NM (2017). A software-defined architecture for control of IoT cyber physical systems. Cluster Computer.

[ref-18] Davoli G, Cerroni W, Tomovic S, Buratti C, Contoli C, Callegati F (2018). Intent-based service management for heterogeneous software-defined infrastructure domains. International Journal of Network Management.

[ref-19] Duran CM, Leal EA, Botero JF (2017). Improving fault tolerance in critical networks through OpenFlow.

[ref-20] Enns R, Bjorklund M, Schoenwaelder J, Bierman A (2011). Network configuration protocol (netconf). https://datatracker.ietf.org/doc/html/rfc6241.

[ref-21] Erickson D (2013). The beacon openflow controller.

[ref-22] Ericsson (2018). 50 Billion Connections 2020. http://www.ericsson.com/thecompany/press/releases/2010/04/1403231.

[ref-23] Farris I, Taleb T, Khettab Y, Song J (2019). A survey on emerging SDN and NFV security mechanisms for IoT systems. IEEE Communications Surveys & Tutorials.

[ref-24] Floodlight (2020). SDN controller. http://www.projectfloodlight.org/floodlight/.

[ref-25] Foerster KT, Schmid S, Vissicchio S (2018). Survey of consistent software-defined network updates. IEEE Communications Surveys & Tutorials.

[ref-26] Fonseca PCda R, Mota ES (2017). A Survey on fault management in software-defined networks. IEEE Communications Surveys & Tutorials.

[ref-27] Gubbi J, Buyya R, Marusic S, Palaniswami M (2013). Internet of things (IoT): a vision, architectural elements, and future directions. Future Generation Computer Systems.

[ref-28] Hakiri A, Berthou P, Gokhale A, Abdellatif S (2015). Publish/subscribe-enabled software defined networking for efficient and scalable IoT communications. IEEE Communications Magazine.

[ref-29] Hakiria A, Gokhale A, Berthou P, Schmidt DC, Gayraud T (2014). Software-defined networking: challenges and research opportunities for future interne. Computer Networks.

[ref-30] Haleplidis E, Hadi Salim J, Halpern JM, Hares S, Pentikousis K, Ogawa K, Wang W, Denazis S, Koufopavlou O (2015). Network programmability with forces. IEEE Communications Surveys Tutorials.

[ref-31] Haque I, Nurujjaman M, Harms J, Abu-Ghazaleh N (2019). Sdsense: an agile and flexible sdn-based framework for wireless sensor networks. IEEE Transactions on Vehicular Technology.

[ref-32] Huang C-Y, Chung W-W, Liu C-Y (2018). SCONN: Design and Implement Dual-Band Wireless Networking Assisted Fault Tolerant Data Transmission in Intelligent Buildings.

[ref-33] Huang C-Y, Wang H-Y, Wu Y-P (2018). SDWDS: fault recovery automation in IoTs.

[ref-34] Jain R, Paul S (2013). Network virtualization and software defined networking for cloud computing: a survey. IEEE Communications Magazine.

[ref-35] Jararweh Y, Al-Ayyoub M, Al-Zoubi D, Benkhelifa E (2018). An experimental framework for future smart cities using data fusion and software defined systems: the case of environmental monitoring for smart healthcare. Future Generation. Computer System.

[ref-36] Javed A, Heljanko K, Buda A, Framling K (2018). CEFIoT: a fault-tolerant IoT architecture for edge and cloud.

[ref-37] Katoh N, Kumar AEds (2011). WALCOM: Algorithms and Computation.

[ref-39] Kreutz DMV, Ramos F, Verissimo P (2013). Towards secure and dependable software defined networks.

[ref-40] Kreutz DMV, Ramos F, Verissimo PE, Rothenberg Ch, Azodolmolky S, Uhlig S (2015). Software-defined networking: a comprehensive survey. Proceedings of the IEEE.

[ref-41] Li G, Wang D, Gallivan T, Doverspike R (2012). On shared risk link group optimization. Journal of Optical Communications and Networking.

[ref-42] Liao Y-Z, Tsai S-C (2018). Fast failover with hierarchical disjoint paths in SDN.

[ref-43] Macedo ELC, de Oliveira EAR, Silva FH, Mello RR, Franca FMG, Delicato FC, de Moraes LFM (2019). On the security aspects of the Internet of Things: a systematic literature review. Journal of Communications and Networks.

[ref-44] Malik A, Aziz B, Al-Haj A, Adda M (2019). Software-defined networks: a walkthrough guide from occurrence to data plane fault tolerance. PeerJ.

[ref-45] Mininet (2020). SDN emulator. https://mininet.org/.

[ref-46] Mohd Aman AH, Yadegaridehkordi E, Attarbashi ZS, Hassan R, Park Y (2020). A survey on trend and classification of internet of things reviews. IEEE Access.

[ref-47] Nunes BA, Mendonca M, Nguyen XN, Obraczka K, Thierry T (2014). A survey of software defined networking: past, present, and future of programmable networks. IEEE Communication survey & tutorial.

[ref-48] Oki E (2012). Linear programming and algorithms for communication networks: a practical guide to network design, control, and management.

[ref-49] OpenDaylight (2020). SDN controller. https://www.opendaylight.org/.

[ref-50] OpenFlow (2014). Switch specification. https://www.opennetworking.org/wpcontent/uploads/2014/10/openflow-spec-v1.3.0.pdf.

[ref-51] OpenFlow (2018). POX Wiki. https://openflow.stanford.edu/display/ONL/POX+Wiki.html.

[ref-52] Pfaff B, Davie B (2013). The open vswitch database management protocol. Informational, Internet Engineering Task Force. http://www.ietf.org/rfc/rfc7047.txt.

[ref-53] Qin Z, Denker G, Giannelli C, Bellavista P, Venkatasubramanian N (2014). A software defined networking architecture for the Internet of Things.

[ref-54] Ramos RM, Martinello M, Esteve Rothenberg C (2013). SlickFlow: resilient source routing in data center networks unlocked by openflow.

[ref-55] Rangisetti AK, Tamma BR (2017). Software defined wireless networks: a survey of issues and solutions. Wireless Personal Communications.

[ref-56] Rehman AU, Aguiar RL, Barraca JP (2019). Fault-tolerance in the scope of software-defined networking (SDN). IEEE Access.

[ref-57] Rehmani MH, Davy A, Jennings B, Assi C (2019). Software defined networks- based smart grid communication: a comprehensive survey. IEEE Communications Surveys & Tutorials.

[ref-58] Savas SS, Tornatore M, Dikbiyik F, Yayimli A, Martel CU, Mukherjee B (2018). RASCAR: recovery-aware switch-controller assignment and routing in SDN. IEEE Transactions on Network and Service Management.

[ref-59] Sgambelluri A, Giorgetti A, Cugini F, Paolucci F, Castoldi P (2013). OpenFlow-based segment protection in ethernet networks. Journal of Optical Communication Networks.

[ref-60] Sinh D, Le L-V, Lin B-SP, Tung L-P (2018). Sdn/nfv—a new approach of deploying network infrastructure for iot.

[ref-61] Softbank Hikari (2013). Protocol oblivious forwarding. http://www.poforwarding.org.

[ref-62] Tayyaba SK, Shah MA, Khan OA, Ahmed AW (2017). Software defined network (SDN) based internet of things (IoT).

[ref-63] Tomovic S, Yoshigoe K, Maljevic I, Radusinovic I (2016). Software-defined fog network architecture for IoT. Wireless Personal Communications.

[ref-64] Wu D, Nie X, Asmare E, Arkhipov D, Qin Z, Li R, Li K (2018). Towards distributed SDN: mobility management and flow scheduling in software defined urban IoT. IEEE Transactions on Parallel and Distributed Systems.

[ref-65] Xia W, Wen Y, Foh CH, Niyato D, Xie H (2015). A survey on software-defined networking. IEEE Communications Surveys and Tutorials.

[ref-66] Yu Y, Li X, Leng X, Song L, Bu K, Chen Y, Yang J, Zhang L, Cheng K, Xiao X (2018). Fault management in software-defined networking: a survey. IEEE Communications Surveys & Tutorials.

[ref-67] Zahmatkesh A, Kunz T (2017). Software defined multihop wireless networks: promises and challenges. Journal of Communications and Networks.

[ref-68] Zhang H, Wang R, Wang H, Wu G (2018). A New lossless fault-tolerance mechanism in hybrid wireless-optical broadband access network. IEEE Access.

[ref-69] Zhang X, Yu S, Zhang J, Xu Z (2018). Forwarding rule multiplexing for scalable SDN-based internet of things. IEEE Internet of Things Journal.

